# Post-translational aging of proteins in osteoarthritic cartilage and synovial fluid as measured by isomerized aspartate

**DOI:** 10.1186/ar2675

**Published:** 2009-04-16

**Authors:** Jonathan B Catterall, Daniel Barr, Michael Bolognesi, Robert D Zura, Virginia B Kraus

**Affiliations:** 1 Department of Medicine, Duke University, 1102 Duke North, Durham, NC 27710, USA; 2 School of Medicine, Duke University, 125 Davison Building, Durham, NC 27710, USA; 3 Department of Surgery, Duke University, 7690 HAFS Building, Hospital North, Durham, NC 27710, USA

## Abstract

**Introduction:**

Aging proteins undergo non-enzymatic post-translational modification, including isomerization and racemization. We hypothesized that cartilage with many long-lived components could accumulate non-enzymatically modified amino acids in the form of isomerized aspartate and that its liberation due to osteoarthritis (OA)-related cartilage degradation could reflect OA severity.

**Methods:**

Articular cartilage and synovial fluid were obtained from 14 randomly selected total knee arthroplasty cases (56 to 79 years old) and non-arthritis cartilage from 8 trauma cases (51 to 83 years old). Paired lesional cartilage and non-lesioned OA cartilage were graded histologically using a modified Mankin system. Paired cartilage and synovial fluids were assayed for isomerized aspartate, phosphate-buffered saline/EDTA (ethylenediaminetetraacetic acid) extractable glycosaminoglycans, and total protein. Macroscopically normal non-lesioned OA cartilage was separated into superficial and deep regions when cartilage thickness was at least 3 mm (n = 6).

**Results:**

Normalized to cartilage wet weight, normal cartilage and deep non-lesioned OA cartilage contained significantly (*P *< 0.05) more isomerized aspartate than superficial non-lesioned OA cartilage and lesioned cartilage. Synovial fluid isomerized aspartate correlated positively (*R*^2 ^= 0.53, *P *= 0.02) and glycosaminoglycans correlated negatively (*R*^2 ^= 0.42, *P *= 0.04) with histological OA lesion severity. Neither synovial fluid isomerized aspartate nor glycosaminoglycans nor total protein correlated with histological scores of non-lesioned areas.

**Conclusions:**

We show for the first time that human cartilage and synovial fluid contain measurable quantities of an isomerized amino acid and that synovial fluid concentrations of isomerized aspartate reflected severity of histological OA. Further assessment is warranted to identify the cartilage proteins containing this modification and to assess the functional consequences and biomarker applications of this analyte in OA.

## Introduction

As proteins age, they undergo non-enzymatic post-translational modifications leading to accumulation of these modifications in long-lived proteins that potentially can alter both their structure and their properties. In the intracellular milieu, non-enzymatic protein modifications can be repaired or the protein replaced [1]. However, in extracellular proteins whose turnover is slow, non-enzymatic modifications can accumulate in a time-dependent manner. This build-up of age-related changes can be used as a biological clock, allowing the ages of proteins to be determined [2,3]. The rate of amino acid modification is influenced by local conditions such as pH [4,5], temperature [6], and protein structure and conformation [7-10] but is also dependent on the amino acid itself [1]. As these changes may bring about structural alterations, they are not necessarily biologically silent as evinced by the association of racemized and isomerized amino acids in human tissues with a variety of disease states, including cataract formation, Paget disease of bone, Alzheimer disease, and UV radiation-induced skin damage [11]. The formation of isomerized aspartate (IsoAsp) within matrix proteins could also interfere with the normal turnover of cartilage as certain proteases, including matrix metalloproteinase-3 and some caspases, are unable to cleave their substrate if an IsoAsp is present in the target sequence [12]. Nothing at present is known of the biological effects of these amino acid changes in cartilage, thus representing a significant knowledge gap.

As part of cartilage homeostasis, cartilage proteins are continually degraded and replaced by the chondrocytes. This matrix turnover happens most rapidly in the vicinity of the cells [13] and is greatly reduced in the interterritorial matrix that is further removed from the cells. The proof of this concept so far has been demonstrated for the proteoglycan component of cartilage [14]. As there is very little protein turnover within the interterritorial matrix, the collagen found within this region is believed to be the longest lived and so the most susceptible to age-related post-translational damage. Turnover also varies with distance from the articular cartilage surface [14], age [15], and between different cartilage matrix molecules. For instance, proteoglycans such as aggrecan turn over much more quickly (3 to 25 years) [2] than the collagen molecules (half-life of 100 to 400 years) [16,17]. In this study, we hypothesized that osteoarthritis (OA)-related cartilage degradation would increase the liberation of aged protein fragments containing IsoAsp from cartilage into synovial fluid and that synovial fluid IsoAsp content would reflect OA-related cartilage turnover and degradation state. To evaluate this hypothesis, we analyzed synovial fluid and matched cartilages from the same individuals as well as normal age-matched cartilages for IsoAsp, protein, and phosphate-buffered saline (PBS)/ethylenediaminetetraacetic acid (EDTA) extractable glycosaminoglycan (GAG) (representing already fragmented and readily solubilized protein components with GAG chains) [18]. We found that synovial fluid levels of IsoAsp reflected severity of cartilage histological degeneration.

## Materials and methods

### Cartilage and synovial fluid collection

**Figure 1:**
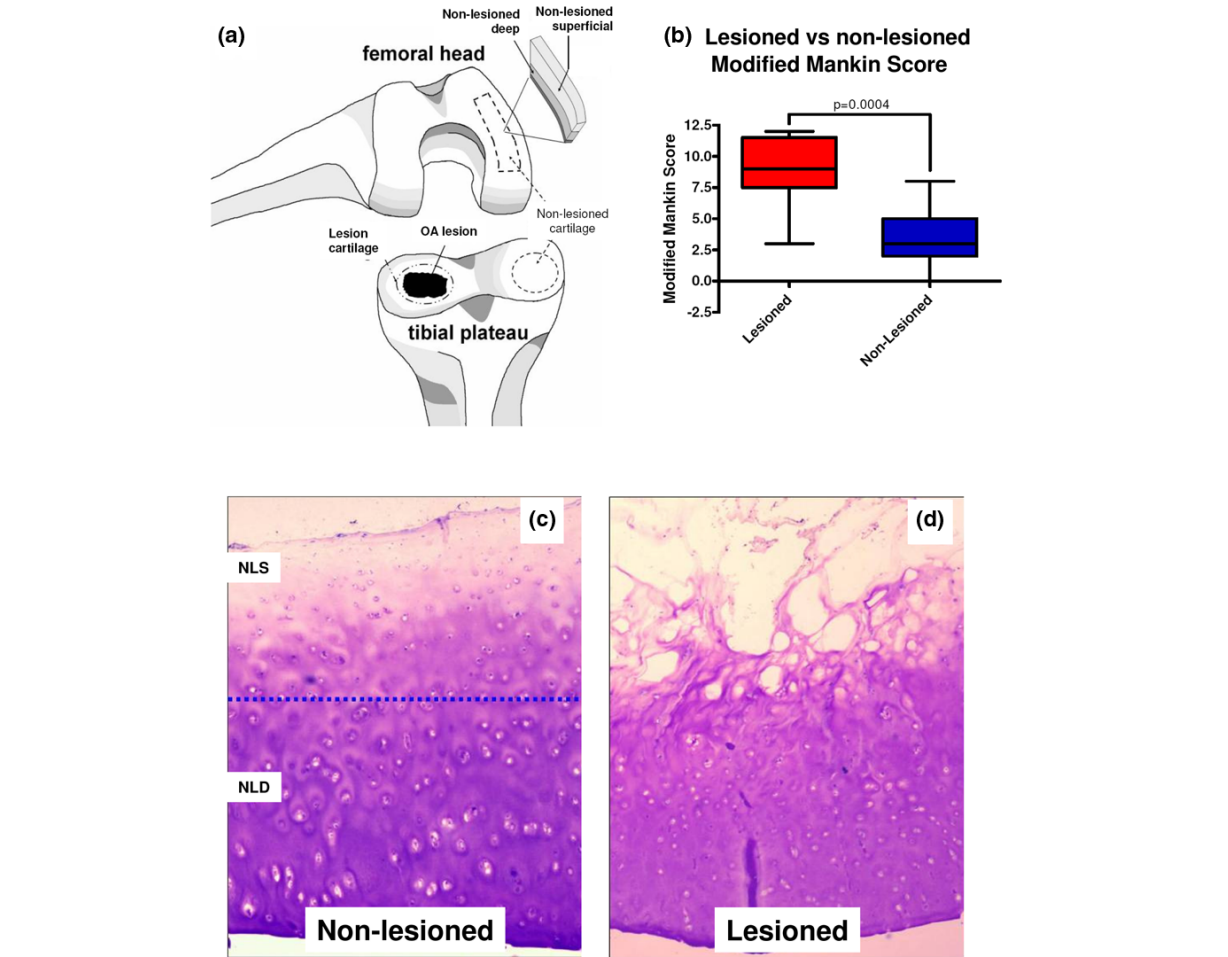
Characterization of cartilage specimens. **(a) **Schematic of the cartilage sampling locations at the lesion and remote from the lesion yielding cartilage from deep and superficial non-lesioned areas. **(b) **Comparison of modified Mankin scores for both lesioned and non-lesioned osteoarthritis (OA) cartilage. Representative toluidine blue-stained cartilage histological sections show the proteoglycan content of **(c) **non-lesioned superficial (NLS) and non-lesioned deep (NLD) OA cartilage and **(d) **lesioned OA cartilage.

Waste articular cartilage and synovial fluid were obtained from 14 cases of randomly selected total knee arthroplasties performed to alleviate symptoms of OA (Table 1). Normal non-arthritic control samples were obtained from trauma patients who showed no signs of OA as determined by the surgeon and macroscopic inspection of the specimens. Samples were collected under Institutional Review Board approval as waste surgical specimens. Cartilage specimens were immediately washed four times with PBS with 0.02% sodium azide (no Ca^2+^, no Mg^2+^) (pH 7.2) (hereafter PBS) to remove body fluids, scored for severity of cartilage degradation according to the Collins grade [19], and cut into strips as either lesioned or non-lesioned OA cartilage based on location and macroscopic appearance (Figure 1a). When non-lesioned OA cartilage was at least 3 mm thick, a section was divided in half lengthwise to produce separate superficial and deep portions of equal thickness (n = 6 of the 14). Strips for histological analysis were painted with 20% (vol/vol) black Indian ink (Sanford Corporation, Bellwood, IL, USA) as previously described [20] to facilitate differentiation of superficial and deep regions. All specimens were processed and frozen at -80°C within 8 hours of surgery. Synovial fluid was centrifuged (8°C, 3,500 *g*, 5 minutes), and the supernatant was aliquoted and frozen at -80°C within 2 hours of surgery. Due to viscosity, all synovial fluid samples were treated with 5 U/mL of the hyaluronan-specific *Streptomyces hyaluronidase *(Sigma-Aldrich, St. Louis, MO, USA) overnight at 37°C prior to further analysis.

**Table 1:** Sample characteristics

Specimen	Age, years	Gender	Collins grade	Modified Mankin grade		Samples	
					
				Lesion	Non-lesioned OA cartilage	Cartilage	Synovial fluid
1	74	Female	4	8	-	L/NL	Yes
2	67	Male	3	10	8	L/NL	Yes
3	69	Male	2	7	3	L/NLS-NLD	Yes
4	56	Female	3	12	3	L/NLS-NLD	No
5	76	Male	4	8	5	L/NL	Yes
6	77	Male	4	-	3	L/NLS-NLD	No
7	59	Male	4	5	2	L/NL	Yes
8	79	Female	4	10	0	L/NL	Yes
9	75	Male	4	8	5	L/NLS-NLD	Yes
10	62	Male	4	12	2	L/NLS-NLD	Yes
11	60	Male	4	12	5	L/NLS-NLD	Yes
12	69	Female	4	3	0	L/NL	Yes
13	66	Female	4	-	2	L/NL	No
14	88	Female	4	11	5	L/NL	No

L, lesion; NL, non-lesioned osteoarthritis cartilage; NLD, deep non-lesioned osteoarthritis cartilage; NLS, superficial non-lesioned osteoarthritis cartilage; OA, osteoarthritis.

### Extraction of highly soluble proteins from cartilage

Extraction of soluble proteins from cartilage was performed with minor modifications according to the method of Vilim and colleagues [21]. Cartilage sections were frozen in liquid nitrogen, pulverized, weighed, and mixed with 1.5 mL of PBS/100 mg pulverized cartilage with 10 mM EDTA and protease inhibitor cocktail (Pierce, Rockford, IL, USA) overnight at 4°C. Quantities of up to 0.12 g of pulverized cartilage were used for extraction. Extracts were cleared by centrifugation (8°C, 15,000 *g*, 30 minutes) and dialyzed in 3,500-kDa cutoff cassettes (Pierce, Rockford, IL, USA) against PBS at 4°C for 24 hours with buffer changes at 2 and 5 hours. Extracts were then frozen at -80°C until further analysis.

### Determination of protein content

Total protein was determined using the commercially available BCA™ Protein Assay Kit in accordance with the instructions of the manufacturer (Pierce, Rockford, IL, USA).

### Determination of isomerized aspartate content

IsoAsp content was measured using the commercially available ISOQUANT^® ^IsoAspartate Detection Kit (Promega Corporation, Madison, WI, USA) in accordance with the radioactive detection protocol of the manufacturer. Of note, this kit enabled detection of two forms of aspartate, isomerized aspartate (L-β-Asp) and racemized aspartate (D-α-Asp), but does not recognize the D-β-Asp racemized form.

### Determination of glycosaminoglycan content

GAG content of cartilage extracts and synovial fluid was quantified by dye-binding assay with dimethylmethylene blue (Sigma-Aldrich) as previously described [22].

### Histological section preparation and evaluation

Cartilage sections, previously stained with Indian ink, were cut into 3-mm-thick pieces and embedded in Tissue Tek OCT Compound (Sakura Finetek USA, Inc., Torrance, CA, USA) using Peel-A-Way Disposable Embedding Molds (Polysciences, Inc., Warrington, PA, USA). Molds were wrapped in aluminum foil and stored at -80°C until cryosectioning (8-μm-thick sections), and sections were stored in standard slide boxes wrapped in aluminum foil. At least one section per specimen was stained with 0.04% toluidine blue dye in 0.1 M sodium acetate at pH 4. Each section was graded blinded to specimen location and pairing using a modified Mankin grading system [23].

### Statistical methods

Statistical analyses were performed using GraphPad Prism version 4.02 (GraphPad Software, Inc., San Diego, CA, USA). To meet assumptions of normality, linearity, and homoscedasticity essential to methods of linear regression modeling, we logarithmically transformed IsoAsp, GAG, and protein values and used the D'Agostino and Pearson omnibus normality test to confirm a normal distribution. Results for different cartilage locations were evaluated using the Wilcoxon signed rank test. All variances quoted are standard errors of the mean.

## Results and Discussion

### Sample characteristics

OA samples were collected from a total of 14 patients at the time of knee joint arthroplasty. The samples originated from a cohort of eight men and six women with a mean (standard deviation) age of 69.8 (8.9) years (range 56 to 88 years: 29% were 56 to 64 years old and 71% were at least 65 years old). Based on Collins grades, the majority of specimens had severe degradation at the lesion site: grade 2 (n = 1), grade 3 (n = 2), and grade 4 (n = 9). Cartilage from the lesion and non-lesioned areas had modified Mankin scores ranging from 3 to 12 and from 0 to 8, respectively (Table 1), with the lesional cartilage having a significantly higher (*P *< 0.0005) mean modified Mankin score (Figure 1b). The six non-lesioned OA cartilage specimens at least 3 mm in thickness had modified Mankin scores ranging from 3 to 5 (individuals 62 ± 9 years old, range 56 to 77 years). We also collected eight surgical waste cartilages from patients with acute joint trauma to serve as age-matched non-arthritic control samples (individuals 69.3 ± 11.3 years, range 51 to 83 years). Representative histological sections from non-lesioned and lesioned OA cartilage regions are presented in Figure 1c, d, demonstrating more intense GAG staining in the deep non-lesioned OA cartilage regions and less intense staining in lesioned cartilage (Figure 1d) compared with the superficial non-lesioned OA cartilage regions.

### Change in isomerized aspartate with age in osteoarthritis and non-osteoarthritis trauma cartilage extracts

**Figure 2:**
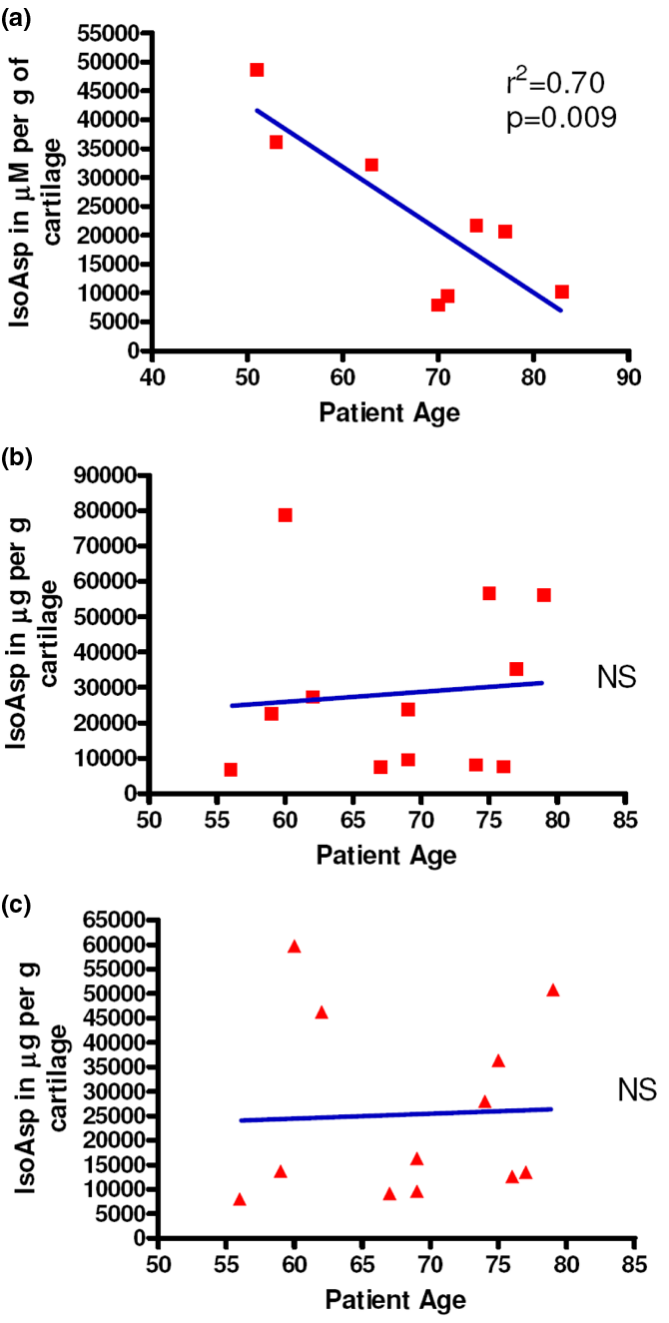
Age relationship of isomerized aspartate (IsoAsp) levels in cartilage extracts. **(a) **IsoAsp levels in cartilage extracts of non-osteoarthritis (non-OA) trauma cartilages. **(b) **IsoAsp levels in cartilage extracts from non-lesioned OA areas. **(c) **IsoAsp levels in cartilage extracts from OA lesion areas. NS, not significant.

To investigate age-related changes in cartilage, we determined IsoAsp levels in cartilage extracts from both OA and non-arthritic trauma cartilage (Figure 2). There was a strong negative correlation of IsoAsp with age (*R*^2 ^= 0.70, *P *= 0.009) in non-arthritic trauma cartilage. There was no association of IsoAsp with age in OA lesion cartilage (*R*^2 ^= 0.008, *P *= 0.78) or non-lesioned OA cartilage (*R*^2 ^= 0.002, *P *= 0.9). The strong negative correlation of IsoAsp with age in non-arthritic cartilage can be explained, as shown previously [24], by the reciprocal rise in these cartilages of the very long-lived racemized aspartate (D-β-Asp) which is not recognized by the protein-isoaspartyl-methyl-transferase (PIMT) assay [24]. We further explored this through an examination of deep and superficial cartilage and synovial fluid content of IsoAsp.

### Isomerized aspartate varied by cartilage location and degeneration status

**Figure 3:**
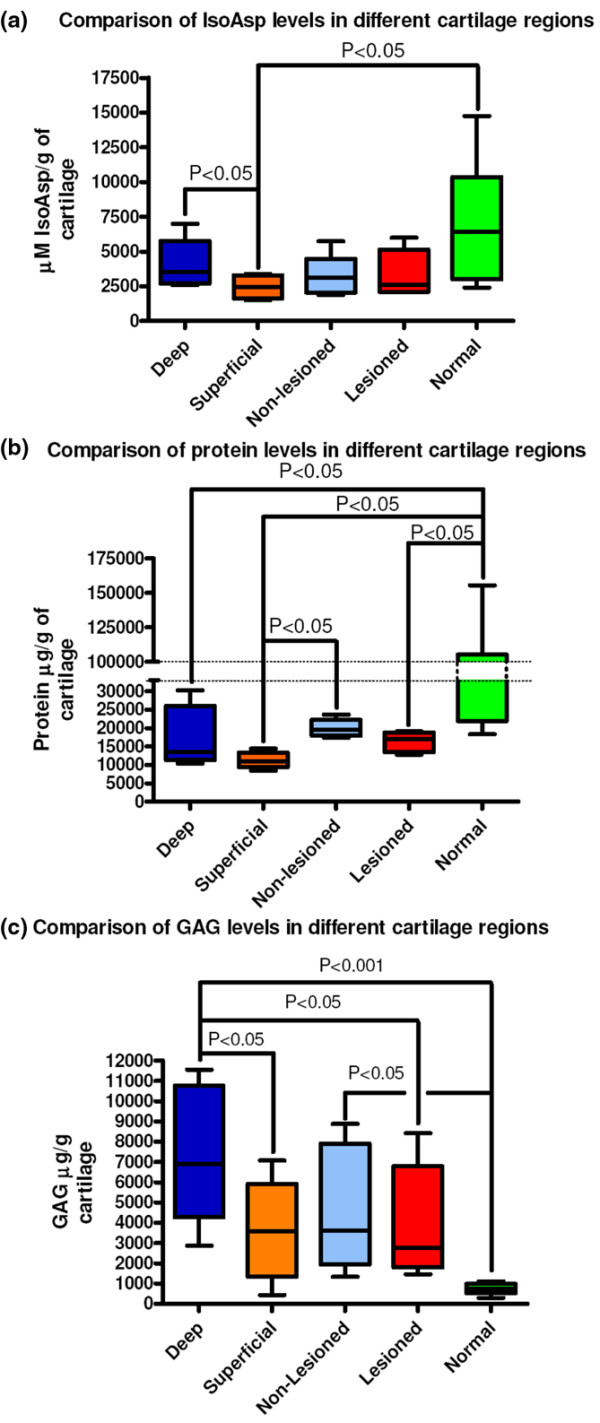
Comparisons of the analyte concentrations in cartilage extract. Mean (standard error of the mean) concentrations of **(a) **isomerized aspartate (IsoAsp), **(b) **protein, and **(c) **glycosaminoglycan (GAG) in different regions within osteoarthritis (OA) cartilage and non-arthritic cartilage. Analytes were normalized to gram of wet weight cartilage. Statistical significance was determined using the Wilcoxon signed rank test, and results represent six individual patients. Using all 14 available cartilage extracts, we found no significant differences between the non-lesional and lesional cartilage extracts but did observe significantly higher (*P *< 0.05) levels of protein in the extracts of non-OA cartilage compared with any OA cartilage samples.

We investigated IsoAsp, GAG, and protein content per cartilage wet weight in the patients (n = 6) for whom we had macroscopically normal OA cartilage obtained remote from the lesion with a minimum cartilage depth of 3 mm. Cartilage extracts were prepared from the deep and superficial halves of these specimens and compared with cartilage extracts prepared from non-OA control cartilages selected prior to extraction to provide the most appropriate age-matched samples (OA 62 ± 9 years of age, n = 6; non-OA 69 ± 11 years of age, n = 8) available. Significantly more IsoAsp and GAG were in the deep non-lesioned OA cartilage compared with the superficial non-lesioned OA cartilage (Table 2 and Figure 3). There was no significant difference between the deep and superficial regions for total protein. OA lesion cartilage was comparable to the superficial OA non-lesioned cartilage for all of the analytes measured and significantly different from deep non-lesioned OA cartilage for IsoAsp (*P *< 0.05). Although differences between superficial and deep regions were apparent, there was no significant difference comparing the overall mean data for all 14 of the OA lesional and matched OA non-lesional cartilages. This result demonstrated that even macroscopically normal appearing OA cartilage is not, on the whole, significantly different than lesional cartilage. We therefore examined normal age-matched specimens of cartilage derived from acute trauma and without pre-existing OA by history or visual inspection. Normal cartilage had higher mean IsoAsp and protein and lower mean concentrations of PBS/EDTA extractable GAG than OA cartilages (Table 2). Significant differences (*P *< 0.001 to 0.05) were found between normal cartilage IsoAsp and superficial non-lesioned OA cartilage, between normal cartilage protein and any OA cartilages, and between normal cartilage GAG and any OA cartilages.

**Table 2:** Mean analyte concentrations in cartilage extracts

Cartilage region	Number	IsoAsp μM/g cartilage ± SEM	Protein μg/g cartilage ± SEM	GAG μg/g cartilage ± SEM
Normal	8	7,092 ± 1,568	65,092 ± 18,017	743 ± 102
Lesioned OA	14	4,942 ± 719	15,281 ± 552^a^	4,463 ± 1,053^b^
Non-lesioned OA	14	7,439 ± 1,273	17,926 ± 1,134^c^	2,911 ± 657^b^
Superficial	6	2,453 ± 315^c^	11,266 ± 818^c^	3,626 ± 921
Deep	6	3,999 ± 665^d^	16,894 ± 3,105^c^	7,316 ± 1,279^a, d, e^

^a^*P *< 0.001 compared to normal cartilage; ^b^*P *< 0.01 compared to normal cartilage; ^c^*P *< 0.05 compared to normal cartilage; ^d^*P *< 0.05 compared to superficial cartilage; ^e^*P *< 0.05 compared to osteoarthritis (OA) lesion cartilage. GAG, glycosaminoglycan; IsoAsp, isomerized aspartate; SEM, standard error of the mean.

### Synovial fluid alterations associated with osteoarthritis lesion severity

**Figure 4:**
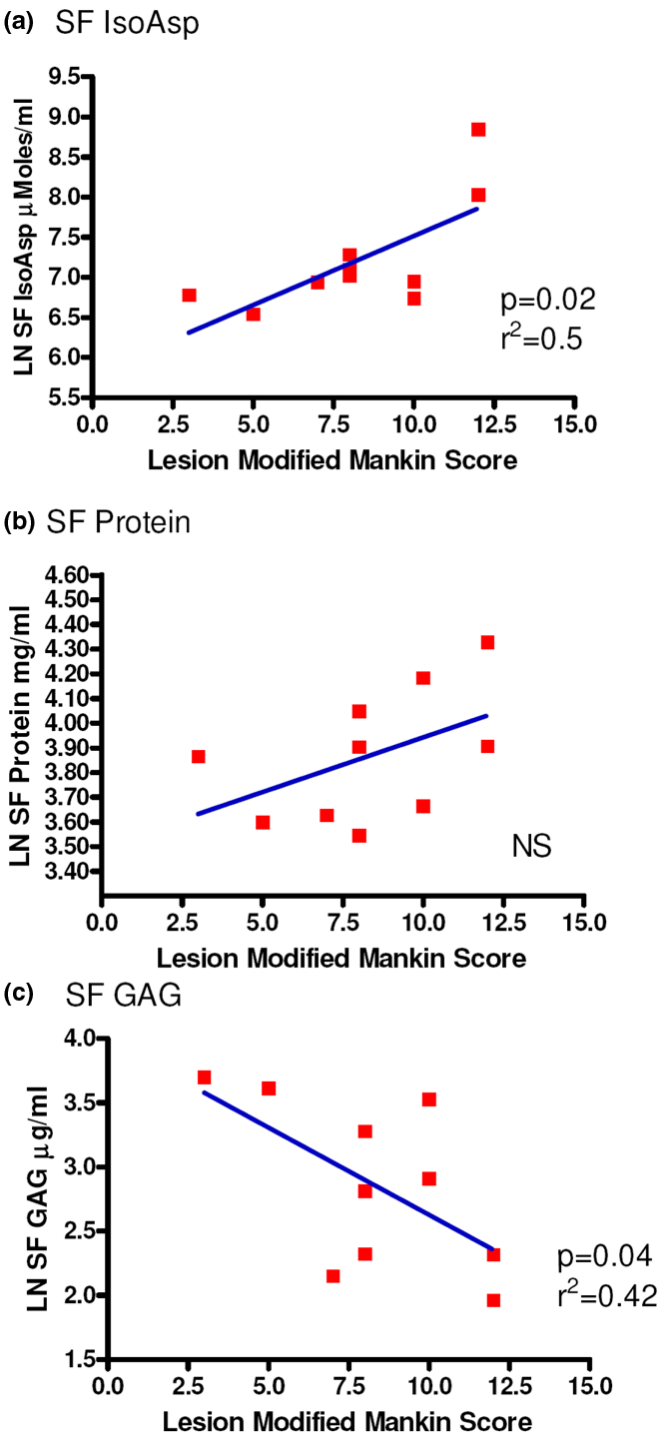
Association of synovial fluid analytes and osteoarthritis severity. Association of synovial fluid **(a) **isomerized aspartate (IsoAsp), **(b) **protein, and **(c) **glycosaminoglycan (GAG) with the modified Mankin scores of lesional cartilage. Analytes were natural logarithm (LN)-transformed to produce a normal distribution as determined by the D'Agostino and Pearson omnibus normality test. Results represent 10 separate individual patients for whom both cartilage and synovial fluid were available (Table 1). NS, not significant; SF, synovial fluid.

To understand the effect of OA lesion on synovial fluid constituents, we measured synovial fluid IsoAsp, protein, and GAG concentrations and compared these values with the amount of damage at the lesion as determined by the modified Mankin score. All synovial fluid values were log-transformed to obtain a Gaussian distribution before linear regression analysis (Figure 4). Synovial fluid IsoAsp concentration demonstrated a significant positive correlation with OA lesional histological severity (*R*^2 ^= 0.40, *P *= 0.039). Total synovial fluid protein showed a similar but non-significant (*R*^2 ^= 0.23, *P *= 0.16) positive correlation with cartilage damage. However, synovial fluid GAG levels decreased significantly with lesion severity (*R*^2 ^= 0.42, *P *= 0.04). These data are in agreement with our PBS/EDTA GAG data as more GAG was extractable from the non-OA cartilage than the lesional cartilage, suggesting that more advanced OA has less GAG available for release into the synovial fluid. There were no significant correlations of these synovial fluid analytes with histological scores for the non-lesioned regions: IsoAsp (*R*^2 ^= 0.029, *P *= 0.66), protein (*R*^2 ^= 0.2, *P *= 0.22), or GAG (*R*^2 ^= 5 × 10^-5^, *P *= 0.996). As for OA cartilage, there was no significant correlation between patient age and levels of IsoAsp (*R*^2 ^= 0.08, *P *= 0.41), protein (*R*^2 ^= 0.01, *P *= 0.80), or GAG (*R*^2 ^= 0.02, *P *= 0.65) (data not shown).

## Conclusions

This study is the first to demonstrate the presence of a modified amino acid, in the form of IsoAsp, in OA-affected human cartilage and synovial fluid and to examine associations between IsoAsp content and the degree of OA-related tissue damage. We found significantly more IsoAsp in normal cartilage and the deeper cartilage zones than the superficial non-lesioned OA cartilage and lesional regions. IsoAsp in cartilage is an intermediate non-enzymatic alteration prior to conversion to racemized aspartate. The PIMT enzyme does not detect the main racemized form (D-β-Asp). Thus, the decline in IsoAsp from a tissue or body fluid could be due to conversion to a racemized form or due to loss from tissue. With these data, we show that it is due to loss from the tissue and in proportion to lesion severity. Our previous work demonstrated that racemization is not increased in OA non-lesioned cartilage relative to normal cartilage [24], which is also supportive of our conclusion that IsoAsp in synovial fluid represents catabolism of molecules of intermediate age. The relative accumulation of IsoAsp in the deep regions of cartilage that was comparable to control cartilage shows, as suspected, that the superficial zone is more actively turning over than the deep region. An alternative explanation may be that IsoAsp accumulates due to steric hindrance from intact matrix and matrix interaction, preventing the conversion of aspartate to the D-β-Asp racemized form. Due to the steady age-related increase in aspartate racemization in normal cartilage previously observed by us and others, we believe this is unlikely [24-26].

The amount of readily extractable GAG from the deep regions of non-lesioned OA cartilage contrasted with the very small amounts of readily extractable GAG from normal cartilage. This suggests that the cartilage in these deep, albeit non-lesioned, areas of an OA cartilage is not normal but rather that there has been previous proteolysis of GAG-bearing proteins that are readily solubilized with the addition of PBS/EDTA alone. Our data are consistent with previous results showing that cartilage aggrecan degradation proceeds in a two-state manner in rheumatoid arthritis [27]. That study showed that, at early stages, the GAG-containing regions are lost from cartilage and the amount of GAG in synovial fluid declines with increasing disease severity. This is exactly the result we find. The authors of that study further found that the hyaluronan-binding (G1) domain of aggrecan is retained early in the disease process and then finally released to synovial fluid and so follows a reciprocal pattern to GAG loss. We did not measure hyaluronan-binding region fragments. We also know that aggrecan plays a protective role in preventing degradation of collagen fibrils [28]. With increasing loss of proteoglycan, there is increasing degradation of collagen and other matrix components [29], and this is entirely compatible with our results showing a tendency to increased protein loss to the synovial fluid with increasing lesion severity and loss of GAG from the cartilage. The fact that IsoAsp also increased with disease severity suggests that the trend to increased synovial fluid protein is not in fact derived from repair but rather primarily from cartilage degradation.

Age-related modifications of lesional cartilage were comparable to superficial non-lesioned OA cartilage. Deep cartilage extracts had more GAG than found in the superficial cartilage, a result corroborated by histological observations. Moreover, the observation that there was reduced GAG present in the lesional extracts was consistent with previous studies [30-34]. All of these data agree in principle with the molecular biology data of Fukui and colleagues [35], who observed that cartilage erosion leads the newly exposed chondrocytes now at the surface to take on the gene expression profiles of the superficial cartilage chondrocytes.

While our study has added significantly to the body of knowledge concerning the effects of OA on human cartilage and synovial fluid contents, several important questions require further investigation. The unique ability of synovial fluid IsoAsp to reflect OA lesion severity makes it an attractive disease marker; however, its full potential cannot be realized without identification of the primary IsoAsp-containing molecule(s). That knowledge would allow the calculation of the modified-to-unmodified molecule ratio and provide a potential marker to facilitate clinical treatment and understanding, analogous to the markers HbA1C in diabetes and αCTX-I/βCTX-1 in Paget disease of bone [36]. Furthermore, the role of IsoAsp in the pathophysiology of OA deserves consideration and further investigation based on the paradigm provided by another form of modifications, racemization, that has been shown to destabilize the collagen triple helix [37].

The data presented here are consistent with our belief that 'aged' neo-epitopes found in the older zones of cartilage have the potential to be important biomarkers of OA. We believe that non-progressive OA represents a balance of catabolic and anabolic processes and that most of the proteins released as part of cartilage turnover will be the recently synthesized proteins, which will have greatly reduced levels of age-related protein modifications such as IsoAsp. However, in active OA progression, catabolism will outpace anabolism and destruction of the deeper cartilage zones will occur, leading to the release of these age-modified proteins. We believe that these aged molecules may have the potential to more accurately predict active progressive cartilage destruction as their levels will be independent of the confounding factor of increased synthesis, which can occur during active repair.

In summary, we have demonstrated for the first time that (a) proteins in the deeper zones of cartilage contain more age-related post-translational modifications in the form of the isomerized amino acid IsoAspartate and (b) synovial fluid levels of the age-related protein post-translational modification IsoAspartate correlated with increased cartilage damage. Our finding that protein modification, specifically IsoAsp, reflects severity of cartilage damage suggests that age-related post-translational protein modifications have the potential to serve as disease activity and progression biomarkers in OA.

## Abbreviations

EDTA: ethylenediaminetetraacetic acid; GAG: glycosaminoglycan; IsoAsp: isomerized aspartate; OA: osteoarthritis; PBS: phosphate-buffered saline; PIMT: protein-isoaspartyl-methyl-transferase.

## Competing interests

The authors declare that they have no competing interests.

## Authors' contributions

JBC and DB contributed equally to the experimental design, the preparation of the manuscript, and the statistical analysis. MB and RDZ coordinated and organized the primary sample collection and critically evaluated both the study design and the manuscript. VBK conceived and designed the study, supervised the project, and assisted in both the statistical analysis and the manuscript preparation and editing. All authors read and approved the final manuscript.
